# Editorial: Diets, Gut Microbiota, and Host Metabolism

**DOI:** 10.3389/fnut.2020.00108

**Published:** 2020-07-22

**Authors:** Jie Yin, Liwei Xie, Yuheng Luo, Helieh S. Oz

**Affiliations:** ^1^College of Animal Science and Technology, Hunan Agriculture University, Changsha, China; ^2^State Key Laboratory of Applied Microbiology Southern China, Guangdong Provincial Key Laboratory of Microbial Culture Collection and Application, Guangdong Open Laboratory of Applied Microbiology, Guangdong Institute of Microbiology, Guangdong Academy of Sciences, Guangdong, China; ^3^Institute of Animal Nutrition, Sichuan Agricultural University, Chengdu, China; ^4^Department of Medicine, University of Kentucky Medical Center, Lexington, KY, United States

**Keywords:** diets, gut microbiota, host metabolism, dysbiosis, metagenomics, metabolomics

Human gut hosts ~100 trillion bacteria, which eventually fall into about 500–1,000 bacterial species. Microbial density and diversity are established very rapidly in the gut of the newborn and drive stabilization of the normal commensal microbiota. However, the gut microbiota diversity and compositions are affected by genetic background, but most importantly by diets and environment (1–3). Wang et al. explored the relationships among diet supplements, gut microbiota, host genetics and metabolic status, leading to the conclusion that diets more intensively disturbed the structure of gut microbiota in excess of genetic change, particularly under leptin deficient conditions. Protein is a major dietary nutrient and Li et al. systemically analyzed the colonic microbiota and metabolic responses to different dietary protein sources in a piglet model. Hu et al. further indicated a role of amino acids in gut microbiota compositions. Starch acts as a major energy source of the daily diet and is the largest fraction among human and monogastric animal diets. Yu et al. demonstrated that the different dietary starch types treatment altered the intestinal microbiota and metabolite profiles of the pigs, and dietary with higher amylose may offer potential benefits for gut health. In an amazonian catfish *Panaque nigrolineatus* model, McDonald et al. discussed the microbiome and predictive metagenomic profiles in response to dietary a wood alone or a less refractory mixed diet containing wood and plant nutrition and a marked change was noticed in the enteric bacterial community composition. Besides, rutin, polysaccharide, matrine, fiber, quercetin, ahlorogenic acid, aloin, pinocembrin, xylooligosaccharide, and other active components have been reported to shape gut microbiota compositions in this Research Topic.

Gut microbiota is also changed in various pathologic conditions and has been considered as an objective measurement for some diseases (4–6). For example, Liu et al. provided a comprehensive understanding of the link between the gut microbiota and insomnia disorder and constructed a prediction model utilizing artificial neural network, which might be used for auxiliary diagnosis of insomnia disorder. Meanwhile, the potential relationships between gut microbiota and childhood leukemia also have been discussed by Wen et al., which may help build a healthy gut microbiota by adjusting the diet construction to deal with childhood leukemia. Thus, manipulation of gut microbiota has been considered a potential target for treating diseases.

Probiotics are live microorganisms that confer health benefits on the host via an improvement of gut microbiota compositions, and metabolism, or directly interacting with host (7, 8). In this Research Topic, several studies focused on the dietary benefits of probiotics. A clinic study showed that co-administration of probiotics with azithromycin is a promising therapy for *Mycoplasma pneumoniae* pneumonia treatment which could prevent and treat antibiotic-associated diarrhea effectively (Liang et al.). In the dextran sulfate sodium-induced colitis model, *Lactobacillus brevis* and *Bacillus amyloliquefaciens* are reported to alleviate colonic damage by reprograming gut microbiota ()Diang et al.; Cao et al.. Constipation is a common gastrointestinal disease, which is characterized by gut dysbiosis. However, *Bifidobacterium* colonization can alleviate constipation more efficiently by improving the water, propionate and butyrate content in feces, and overall gastrointestinal transit time (Wang et al.). In animal industry, probiotics are widely introduced to improve health and growth. Xu et al. reported that *Lactobacillus paracasei subsp. paracasei* L1 possesses probiotic properties (i.e., adhesion, aggregation, hydrophobicity, as well as survival upon exposure to various gastrointestinal conditions, and lack hemolytic and decarboxylation activities) and improves the growth performance in chickens. In weaning piglets, dietary *Lactobacillus plantarum* PFM 105 promotes intestinal development (intestinal villi) through modulation of gut microbiota and metabolism (Wang et al.). Similarly, Hu et al. probiotic *Bacillus amyloliquefaciens* has been indicated as an alternative of antibiotics on digestive enzymes activity and intestinal integrity of piglets.

We also received several articles about probiotic isolation. Somashekaraiah et al. isolated seven potential of lactic acid bacteria with the best probiotic attributes from the sap extract of the coconut palm inflorescence-Neera. Gong et al. also isolated a new *Pediococcus pentosaceus strain (SL001)* from soil samples and found it can be used as a dietary probiotic in freshwater fish aquaculture by enhancing immunity and promoter growth rate of grass carps.

It is clear from these papers in this Research Topic that the field of diet-gut microbiota-host metabolism interplay has many avenues to explore. The impact of dietary nutrients (i.e., protein, starch, fiber, amino acids, probiotics, and other active substances) on the gut microbiome is currently an area of intense investigation and hopefully will lead a detailed understanding of how specific nutrient can influence the ecology of the gut microenvironment to produce metabolic benefits. Meanwhile, gut microbiota dysbiosis is associated with various kinds of diseases, thus manipulation of gut ecology has been widely incorporated into clinical methods. Bacterial metabolites, microRNA, bacteriocin, and microbiota sensing pathways are increasingly recognized to be involved in the diet-gut microbiota-host metabolism interplay, while the detailed mechanisms in different models are far from clear.

## Author Contributions

All authors listed have made a substantial, direct and intellectual contribution to the work, and approved it for publication.

## Conflict of Interest

The authors declare that the research was conducted in the absence of any commercial or financial relationships that could be construed as a potential conflict of interest.
